# Identification of potential biomarkers associated with oxidative stress in the pathogenesis of pre-eclampsia

**DOI:** 10.1097/MD.0000000000041784

**Published:** 2025-03-07

**Authors:** Xuejing Liu, Yueting Bai, Han Chen, Nianfeng Qian, Lina Wu, Li Zhao, Shuo Wang, Chong Shen, Hongqing Jiang

**Affiliations:** a Department of Gynecology and Obstetrics, Haidian Maternal and Child Health Hospital, Beijing, China; b Department of Urology, The Second Hospital of Tianjin Medical University, Tianjin, China.

**Keywords:** bioinformatic analysis, biomarker, oxidative stress, pre-eclampsia

## Abstract

Pre-eclampsia (PE) is a multisystem pregnancy disorder characterized by placental and maternal endothelial dysfunction, and affects approximately 5% to 7% of pregnancies worldwide, leading to significant maternal and neonatal morbidity and mortality. Mounting evidence indicates that placental oxidative stress (OS) plays a critical role in the pathogenesis of PE. However, the specific mechanisms associated with OS during the occurrence and progression of PE remain largely unknown. Thus, we aimed to identify the key molecules associated with OS and explore their potential mechanisms in PE. Transcriptome data were downloaded from the Gene Expression Omnibus database, including 80 PE and 77 normal placental tissues. OS-related genes were identified using the Gene Ontology database. Gene Ontology and Kyoto Encyclopedia of Genes and Genomes enrichment analyses were performed to analyze the functions and pathways of the OS-related differentially expressed genes (OS-DEGs). Protein–protein interaction networks were constructed using the Search Tool for the Retrieval of Interacting Genes database, and hub genes were screened using molecular complex detection and CytoHubba. Finally, the diagnostic value and drug-gene interactions of the hub genes were evaluated. We identified 470 differentially expressed genes and 43 OS-DEGs. These genes were mainly enriched in OS-related biological processes, the HIF-1 and MAPK signaling pathways. Furthermore, 5 hub genes were identified: VEGFA, CCL2, mitogen-activated protein kinase 8 (MAPK8), HMOX1, and Cytochrome B-245 Beta Chain (CYBB). CYBB and MAPK8 had the highest diagnostic accuracies, with area under the curve values of 0.767 and 0.764, respectively. We predicted 43 potentially targeted drugs for PE treatment. CYBB and MAPK8 may be valuable biomarkers that mediate OS through multiple pathways to promote the occurrence and development of PE. We conclude from our study that OS has involvement in PE, and improved our understanding of OS-related molecular pathways in the pathogenesis of PE.

## 1. Introduction

Pre-eclampsia (PE) is a pregnancy-related progressive disorder involving multiple organ systems.^[1]^ It remains the leading cause of maternal and fetal morbidity and mortality worldwide and represents a significant public health concern.^[2]^ Globally, there are nearly 76,000 maternal deaths and 500,000 fetal and newborn deaths per year, and PE complicates 5% to 7% of all pregnancies.^[3]^ At present, a significant proportion of women are severely affected during initial diagnosis and even require multidisciplinary joint treatment owing to the complex pathogenesis and heterogeneity of the clinical presentation.^[4]^ Antihypertensive and antispasmodic drugs are mainly used to treat symptoms, and the only treatment option for women with PE is to deliver the fetus and placenta, regardless of their gestational age.^[5]^ No known treatments or therapeutic interventions are capable of slowing down disease progression.^[6]^ Therefore, the identification of new molecules and therapeutic targets for this deadly disease has important clinical implications.

An increasing number of studies demonstrate that oxidative stress (OS) results from an imbalance of reactive oxygen species (ROS) and antioxidant capacity plays an important role in various pathophysiological processes of PE.^[7]^ The 2-stage paradigm is an effective model to determine the pathogenesis of PE.^[8]^ Stage 1 involves abnormal placental implantation and mal placentation, which are strong inducers of OS. Moreover, OS participates in stage 2 maternal endothelial dysfunction and the systemic inflammatory response by stimulating the release of anti-angiogenic factors, pro-inflammatory cytokines, and soluble endoglin into the maternal circulation.^[9]^ However, the underlying molecular mechanisms associated with OS in PE occurrence and progression remain unclear.

We initially used bioinformatics to identify OS-related differentially expressed genes (OS-DEGs) in the PE and performed Gene Ontology (GO) and Kyoto Encyclopedia of Genes and Genomes (KEGG) pathway enrichment analyses of all OS-DEGs. In addition, the interaction association of proteins encoded by differentially expressed genes (DEGs) was explored by constructing protein-protein interaction (PPI) networks. Five hub genes were identified using molecular complex detection (MCODE) and 9 topological methods of CytoHubba. Finally, the correlation between the expression of these 2 key hub genes and their clinicopathological features was analyzed. Importantly, 43 potential targeted drugs were predicted for PE. Our study may help further elucidate the molecular mechanisms underlying the pathogenesis of PE and provide a novel perspective for its diagnosis and treatment.

## 2. Materials and methods

### 2.1. Study design

**Figure 1.**
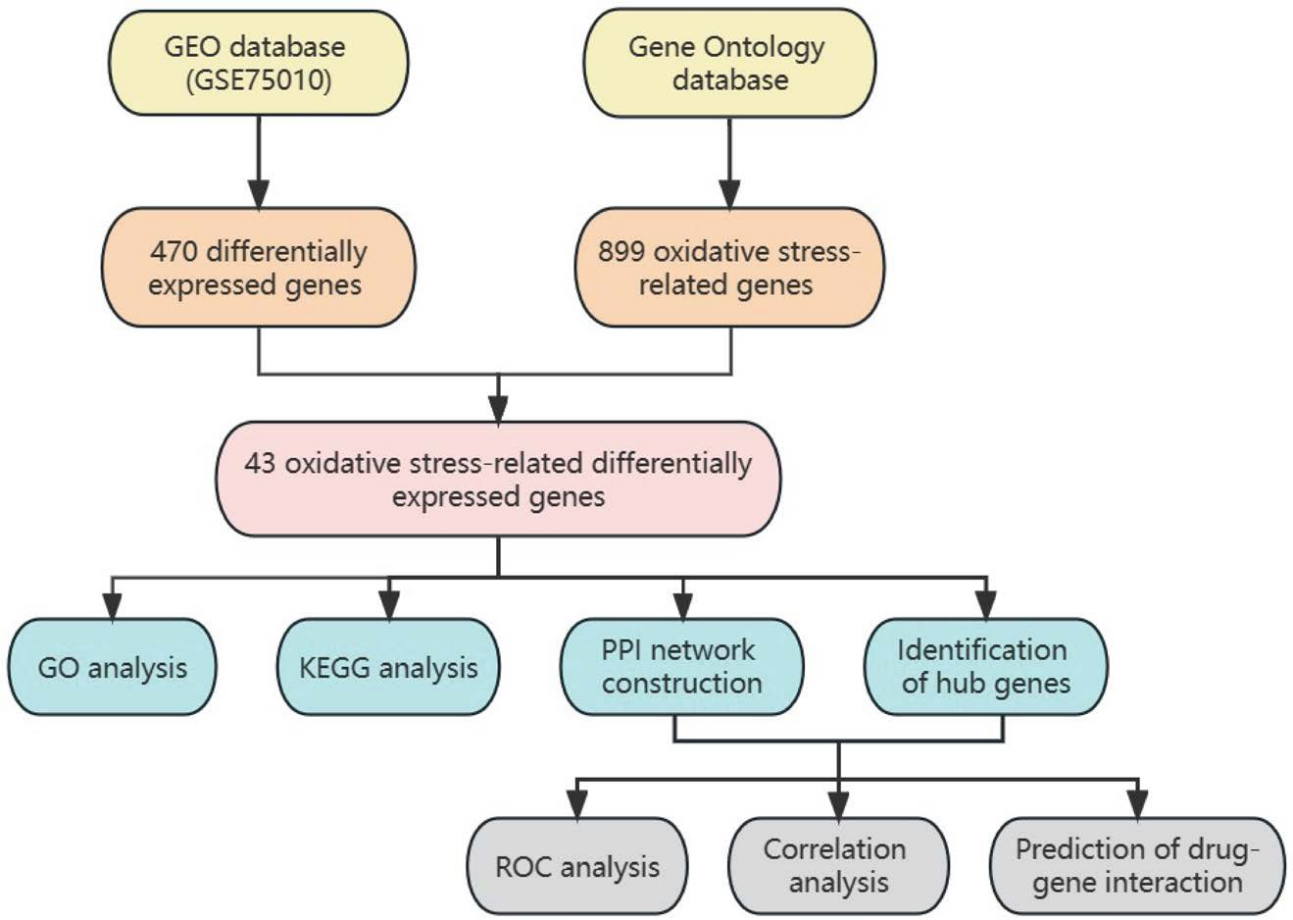
Flow chart of our study. GEO = Gene Expression Omnibus, GO = Gene Ontology, KEGG = Kyoto Encyclopedia of Genes and Genomes, PPI = protein–protein interaction, ROC = receiver operating characteristic.

The workflow of our study is depicted in Figure 1. Transcriptome data were downloaded from the Gene Expression Omnibus (GEO) database, OS-related genes were identified using the GO database, and various analyses were performed to evaluate the functions, pathways, and interactions of the identified genes.

### 2.2. Data collection and preprocessing

Gene expression profiles and corresponding clinical data of PE were downloaded from the GEO database (http://www.ncbi.nlm.nih.gov/geo; accession number: GSE75010). This dataset was established on the GPL6244 platform (Affymetrix Human Gene 1.0 ST Array) and consisted of 80 PE samples and 77 normal control placental samples. Probes were converted to their respective gene symbols, and multiple probes corresponding to the same gene were randomly selected with the highest variance based on the annotation information on the platform. Finally, the gene expression matrix was constructed.

### 2.3. Screening of DEGs and OS-DEGs

The limma R package was used with threshold values of the adjusted *P* value < .05 and |log fold change (FC)| > 0.265 to screen DEGs between PE and the control groups. Principal component analysis and volcano plots of DEGs were visualized using the ggplot2 package in R. OS-related genes were downloaded from the GO database (http://geneontology.org/) and non-*Homo sapiens* genes were excluded. Overlapping genes from DEGs and OS-related genes were considered OS-DEGs between the PE samples and normal samples. A Venn diagram was drawn using the ggplot2 R package, and heat maps of the OS-DEGs were generated using the ComplexHeatmap R package.^[10]^

### 2.4. Gene Ontology and KEGG enrichment analysis of OS-DEGs

GO and KEGG functional enrichment analyses were performed using the ClusterProfiler package to explore the potential mechanisms of these OS-DEGs. An adjusted *P* value < .05 and count ≥ 2 were considered cutoff values using the human genome as a background reference. A bubble diagram was used to visualize the results of the enrichment analysis using ggplot2. Furthermore, the function in the ClusterProfiler package was utilized to display the genes enriched in GO processes with the smallest *P* value for biological process (BP), cellular component (CC), and molecular function (MF). Chord diagrams of genes related to specific GO annotations were created using GoPlot software.^[11]^

### 2.5. Construction of the PPI network and selection of hub genes

The Search Tool for the Retrieval of Interacting Genes (STRING) database (https://string-db.org/) was used to construct a PPI network of OS-DEGs among the proteins with confidence scores > 0.4.^[12]^ The most densely connected modules were identified from the PPI network using the MCODE plug-in of Cytoscape for further analysis using a degree cutoff of 2, node score of 0.2, *k* core of 2, and a maximum depth of 100. Only modules with scores of >5 were selected. The CytoHubba plug-in in Cytoscape was used to filter the top 10 hub genes in 9 ways: MCC, MNC, degree, EPC, BottleNeck, closeness, radiality, betweenness, and stress. Finally, the intersection of the 9 methods and the MCODE algorithm was used to identify the hub genes.

### 2.6. Potential drugs for the hub genes

The Drug–Gene Interaction Database (DGIdb, http://www.dgidb.org) was used to identify potential druggable targets based on lists of mutations and altered genes. We searched DGIdb to identify new potential drugs that interact with the 2 hub genes and visualized the network using Cytoscape software.

### 2.7. Statistical analysis

All analyses were completed using the R programming language (version 4.1.2) and its relevant packages. The Wilcoxon test was used to compare differences between variables. A receiver operator characteristic (ROC) curve was plotted to evaluate the diagnostic accuracy of the hub genes for PE. Pearson’s correlation analysis was performed to evaluate the relationship between the clinical characteristics and gene expression. A 2-sided *P* < .05 was considered statistically significant.

## 3. Results

### 3.1. Identification of OS-DEGs in pre-eclamptic placental samples

**Figure 2.**
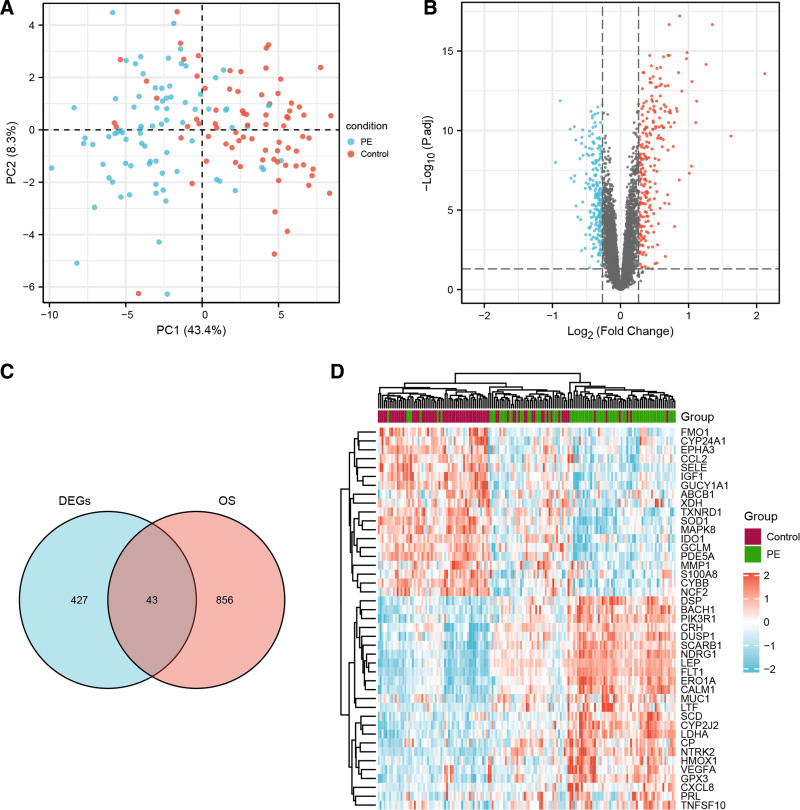
Screening of OS-DEGs. (A) PCA shows an obvious difference between the PE group and the control group. (B) Volcano plot of all DEGs in the PE group and the control group. Red dots represent upregulated genes, blue dots represent downregulated genes, and gray dots represent non-significant genes. (C) Venn diagram of the intersection with DEGs and OS-related genes. (D) Heatmap for hierarchical clustering of 43 OS-DEGs in the control group and the PE group. DEGs = differentially expressed genes, OS-DEGs = oxidative stress-related differentially expressed genes, PCA = principal component analysis, PE = pre-eclampsia.

Principal component analysis of the GSE75010 dataset showed that the PE samples were segregated from the control samples, confirming an excellent intergroup distribution (Fig. 2A). Differential gene expression analysis identified 470 DEGs (247 upregulated and 223 downregulated genes, Table S1, Supplemental Digital Content, http://links.lww.com/MD/O478), that visualized using a volcano plot (Fig. 2B). Subsequently, 899 OS-related genes were downloaded from the GO database (Table S2, Supplemental Digital Content, http://links.lww.com/MD/O478) and intersected with DEGs from GSE75010 to identify OS-DEGs. The Venn diagram shows that 43 genes crossed between DEGs and OS-related genes in the placenta of PE patients (Fig. 2C and Table 1). The top 10 upregulated genes with the smallest *P* value in the PE and control groups were LEP, FLT1, CRH, CP, NDRG1, NTRK2, ERO1A, MUC1, PRL, and LTF. The top ten downregulated genes were MMP1, S100A8, SOD1, IDO1, Cytochrome B-245 Beta Chain (CYBB), GCLM, PDE5A, IGF1, GUCY1A1, and ABCB1. A heat map was prepared to depict the 24 most significantly upregulated genes and 19 downregulated genes (Fig. 2D).

**Table 1:** Forty three **OS-DEGs in pre-eclampsia human placental tissues.**

OS-DEGs	Gene symbol
Upregulated	LEP FLT1 CRH CP NDRG1 NTRK2 ERO1A MUC1 PRL LTF CYP2J2 CXCL8 SCD LDHA DUSP1 SCARB1 VEGFA CALM1 BACH1 GPX3 PIK3R1 HMOX1 DSP TNFSF10
Downregulated	MMP1 S100A8 SOD1 IDO1 CYBB GCLM PDE5A IGF1 GUCY1A1 ABCB1 TXNRD1 CCL2 SELE CYP24A1 MAPK8 EPHA3 FMO1 NCF2 XDH

OS-DEGs = oxidative stress-related differentially expressed genes.

### 3.2. Functional enrichment and pathway analysis of OS-DEGs

**Figure 3.**
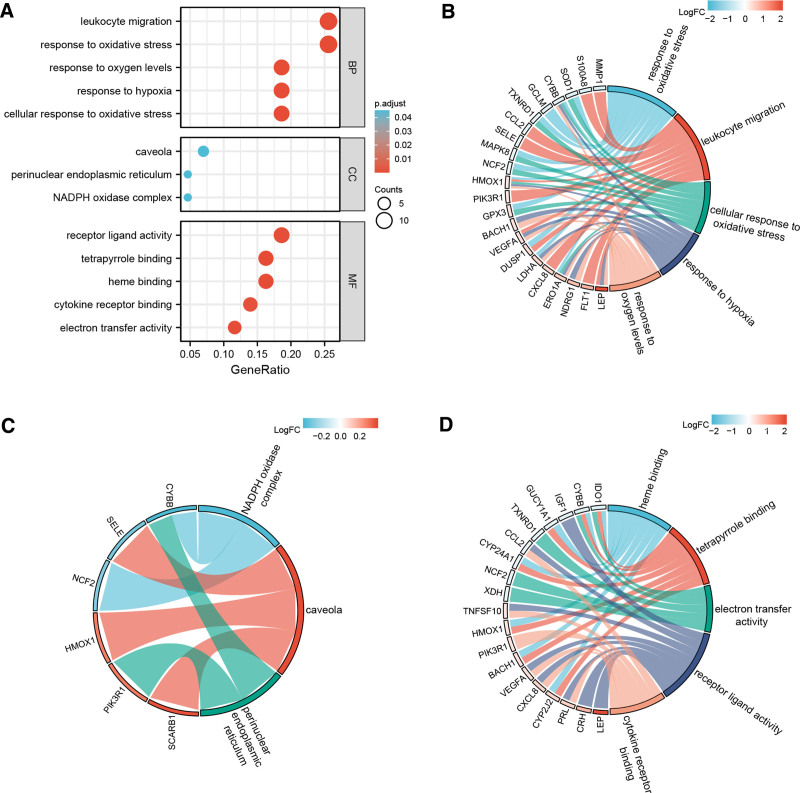
GO enrichment analysis of OS-DEGs. (A) The bubble plots show the processes enriched by OS-DEGs in BP, CC, and MF. (B–D) The chord graph shows the OS-DEGs enriched in the GO categories of BPs, CCs, and MFs, respectively. The color of the fills represents the corresponding logFC values, the size of the fills represents the number of the genes it includes. BP = biological process, CC = cellular component, GO = Gene Ontology, MF = molecular function, OS-DEGs = OS-related differentially expressed genes.

**Figure 4.**
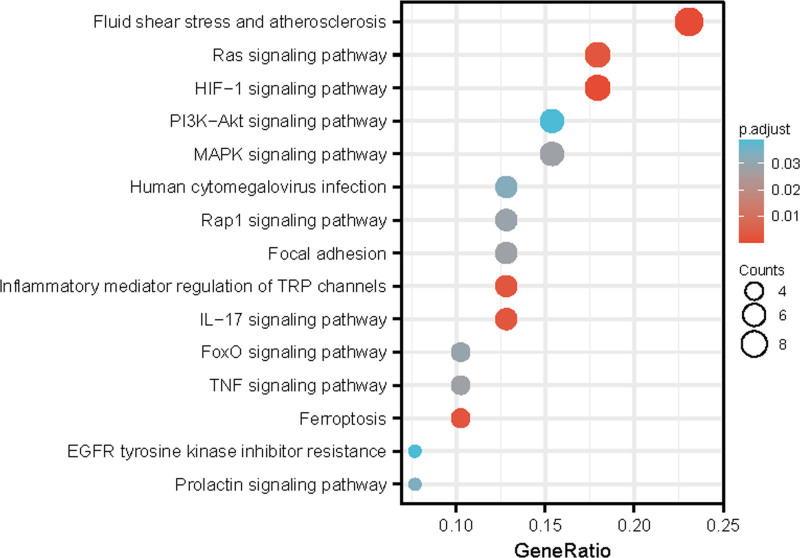
The top 15 KEGG pathway enrichment map of OS-DEGs. KEGG = Kyoto Encyclopedia of Genes and Genomes, OS-DEGs = OS-related differentially expressed genes.

GO analysis, performed to further investigate the underlying biological functions of these OS-DEGs results, for BP, suggested that they were mainly enriched in response to OS, cellular response to OS, and response to oxygen levels. The most enriched “CC” terms were caveola, NADPH oxidase complex, and perinuclear endoplasmic reticulum. The “MF” group mainly contained receptor ligand activity, cytokine receptor binding, and electron transfer activity (Fig. 3A and Table S3, Supplemental Digital Content, http://links.lww.com/MD/O478). Furthermore, chord diagrams were created to display the genes enriched in GO processes with the smallest *P* value for BP, CC, and MF (Fig. 3B–D). Among these, CYBB, CCL2, SELE, HMOX1, mitogen-activated protein kinase 8 (MAPK8), VEGFA, CXCL8, LEP, and PI3KR1 were enriched in multiple BPs. KEGG analysis revealed that the OS-DEGs were highly involved in RAS-, HIF-1-, PI3K-Akt-, MAPK-, and prolactin signaling pathways (Fig. 4 and Table S4, Supplemental Digital Content, http://links.lww.com/MD/O478).

### 3.3. Establishing the PPI network of OS-DEGs and identifying hub genes

**Figure 5.**
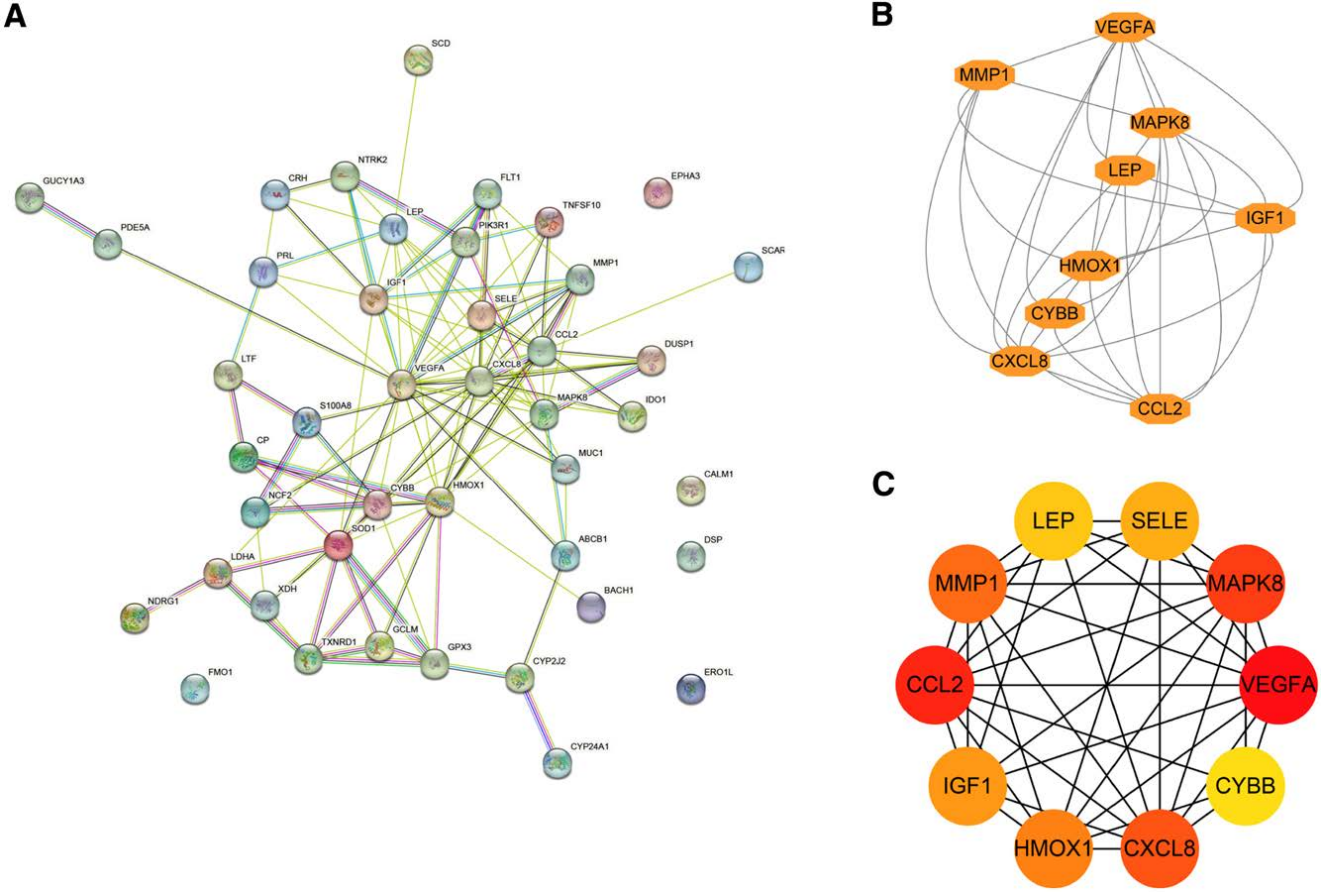
Construction of the PPI network of OS-DEGs and screening of the hub genes. (A) The PPI network of OS-DEGs was downloaded from the STRING database, with 43 nodes and 119 edges. (B) The key module with the highest score constructed by the MCODE plug-in in Cytoscape consists of 9 genes. (C) The top 10 hub genes distinguished using color shading from yellow to red, according to the score. PPI = protein–protein interaction, OS-DEGs = OS-related differentially expressed genes. STRING = Search Tool for the Retrieval of Interacting Genes, MCODE = molecular complex detection.

A PPI network, constructed using the STRING database, contained 43 OS-DEGs (Fig. 5A). The network diagram consisted of 43 nodes and 119 edges. The darker the color and wider the edge, the stronger the evidence supporting the interaction between proteins. Analysis with the MCODE plug-in in Cytoscape revealed that only 1 module had a score > 5, consisting of 9 genes (Fig. 5B). Five hub genes were identified in all MCODE and 9 topological methods of the CytoHubba methods: CYBB, MAPK8, VEGFA, CCL2, and HMOX1 (Fig. 5C and Table 2).

**Table 2:** The top 10 OS-DEGs by MCODE and 9 topological methods of Cytohubba.

MCODE	MCC	MNC	Degree	EPC	BottleNeck	Closeness	Radiality	Betweeness	Stress
CXCL8	VEGFA	VEGFA	VEGFA	VEGFA	HMOX1	VEGFA	VEGFA	VEGFA	HMOX1
MMP1	CCL2	MAPK8	HMOX1	MAPK8	SELE	HMOX1	HMOX1	HMOX1	VEGFA
CCL2	MAPK8	HMOX1	MAPK8	CXCL8	VEGFA	MAPK8	MAPK8	SOD1	SOD1
IGF1	CXCL8	CXCL8	CXCL8	CCL2	MAPK8	CXCL8	CXCL8	GPX3	MAPK8
HMOX1	MMP1	CCL2	CCL2	HMOX1	CCL2	CCL2	CCL2	MAPK8	GPX3
MAPK8	HMOX1	IGF1	IGF1	IGF1	SOD1	IGF1	SOD1	CCL2	CYBB
CYBB	IGF1	SOD1	SOD1	SOD1	CYBB	SOD1	IGF1	CYBB	CCL2
LEP	SELE	CYBB	CYBB	LEP	GPX3	CYBB	CYBB	LEP	LEP
VEGFA	LEP	LEP	LEP	CYBB	LEP	SELE	SELE	IGF1	IGF1
	CYBB	MMP1	SELE	SELE	CXCL8	LEP	MMP1	SELE	CXCL8

MCODE = Molecular Complex Detection, OS-DEGs = oxidative stress-related differentially expressed genes.

### 3.4. Diagnostic accuracy analysis of hub genes and correlation with the uterine pulse index (PI) for PE

**Figure 6.**
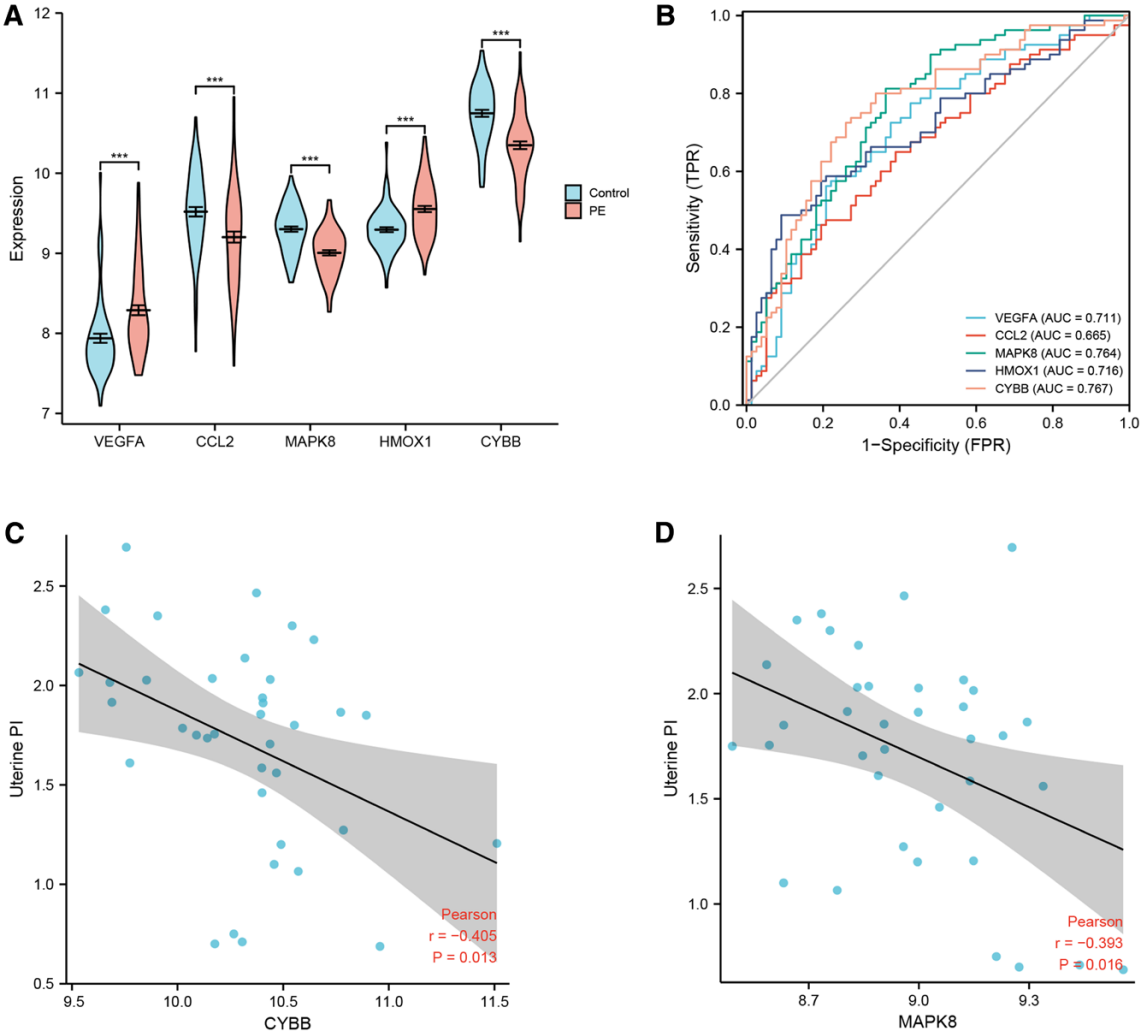
Diagnostic accuracy analysis of candidate 5 hub genes and correlation with uterine PI in the placenta of PE. (A) Violin plot showing expression levels of 5 hub genes in control and PE groups. (B) The ROC curve analysis of hub genes for the diagnosis of PE. (C) Pearson correlation plots between CYBB and uterine PI. (D) Pearson correlation plots between MAPK8 and uterine PI. *P < .05, **P < .01, ***P < .001, ****P < .0001. AUC = the area under the curve, CYBB = Cytochrome B-245 Beta Chain, MAPK8 = mitogen-activated protein kinase 8, PI = pulse index, PE = pre-eclampsia, ROC = receiver operating characteristic, 95% CI = 95% confidence interval.

CYBB, CCL2, and MAPK8 hub genes were specifically downregulated in the PE group, whereas VEGFA and HMOX1 hub genes were significantly increased compared to the control group (Fig. 6A). ROC analysis, used to evaluate the potential diagnostic ability of hub genes to discriminate PE patients from healthy pregnant women, revealed the area under the curve (AUC) values for CYBB, MAPK8, HMOX1, VEGF-A, and CCL2 to be 0.767, 0.764, 0.716, 0.711, and 0.665, respectively (Fig. 6B). Thus, CYBB and MAPK8 were selected as hub genes. In addition, Pearson’s correlation analysis revealed a significant correlation between the uterine PI, CYBB (*r* = −0.405, *P* = .013), and MAPK8 (*r* = −0.393, *P *= .016; Fig. 6C, D).

### 3.5. Predicting potential drugs of hub genes

**Figure 7.**
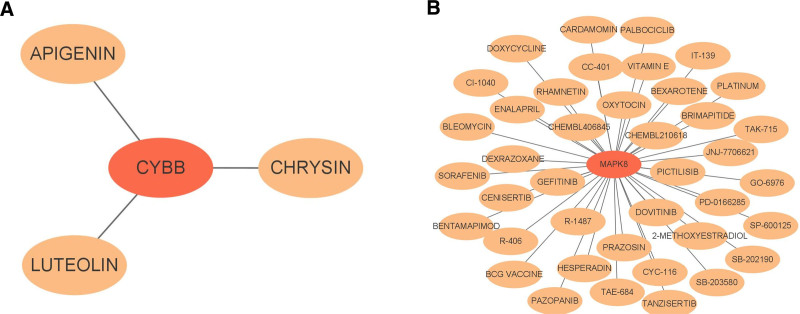
Drug-hub genes interactions. Red rectangles represent hub genes, and orange rectangles indicate potential drug targets of the hub genes. (A) Potential drugs corresponding to CYBB. (B) Potential drugs corresponding to MAPK8. CYBB = Cytochrome B-245 Beta Chain, MAPK8 = mitogen-activated protein kinase 8.

The Drug–Gene Interaction Database (DGIdb) analysis showed that apigenin, chrysin, and luteolin interacted with CYBB (Fig. 7A). Furthermore, 40 drugs (including tanzisertib, brimapitide, and cardamomin) regulated MAPK8 (Fig. 7B and Table S5, Supplemental Digital Content, http://links.lww.com/MD/O478). These results suggest that these targeted drugs may be effective in treating PE patients.

## 4. Discussion

PE is associated with a 3- to 25-fold increase in maternal complications, such as eclampsia, HELLP syndrome, acute kidney injury, disseminated intravascular coagulation, pulmonary edema, placental abruption, and death.^[13,14]^ Additionally, fetal risks include restricted intrauterine growth, low birth weight, preterm birth, and fetal demise.^[15]^ Numerous studies show that placental hypoxia caused by inadequate uterine spiral arteriolar remodeling is central to its pathogenesis.^[16]^ OS is a hallmark of PE and plays a key role in the defective placental development at the maternal-fetal interface.^[17]^ However, the specific mechanisms associated with OS during the occurrence and progression of PE remain largely unknown.

A comparison of the DEGs with OS-related gene lists intersected 43 OS-DEGs, including 24 upregulated genes and 19 downregulated genes, while the GO results demonstrated that the OS-DEGs were mainly involved in the response to OS, cellular response to OS, and response to oxygen levels. Most of these genes acted on the caveola, the NADPH oxidase complex, and the perinuclear endoplasmic reticulum. These results indicate that OS may be involved in the pathogenesis of PE. High levels of systemic OS in pregnant women can cause placental dysfunction and PE. Extensive studies indicated that pre-eclamptic placental samples showed an imbalance between ROS production and endogenous antioxidant mechanisms initiated by repetitive ischemia-reperfusion injuries.^[18]^ Our results are consistent with these findings. GO chord diagrams showed that CYBB, CCL2, SELE, HMOX1, MAPK8, VEGFA, CXCL8, LEP, and PI3KR1 were enriched in at least 2 of these 3 aspects: BP, CC, and MF, indicating that these genes may play an important role in PE. KEGG results showed that the target proteins were highly enriched in the RAS signaling pathway, HIF-1 signaling pathway, PI3K-AKT signaling pathway, and MAPK signaling pathway. The RAS signaling pathway involves multiple signal transduction pathways that contribute to cell proliferation, invasion, migration, and inhibition of apoptosis.^[19]^ The circulating renin-angiotensin-aldosterone system and placental RAS are activated in the placenta in PE patients.^[20]^ MEG3 activates the RAS pathway by inhibiting RASA1 expression resulting in a limited oxygen supply owing to placental hypoperfusion that leads to HIF-mediated hypoxia-mediated genome reprogramming. The PI3K-AKT signaling pathway mediates diverse cellular regulatory processes including cell growth, proliferation, metabolism, and migration.^[21]^ Transfer of miR-15a-5p by placental exosomes promoted PE progression by regulating the PI3K/AKT signaling pathway.^[22]^ The MAPK signaling pathway is activated by cytokines, growth factors, and OS, and participates in regulation of the cell cycle, cellular differentiation, and apoptosis.^[23]^ Inadequate uteroplacental blood perfusion can induce the development of a generalized maternal systemic inflammatory response to activate the MAPK pathway in the placental villi. Recent studies indicate that hydrogen suppresses OS by inhibiting the MAPK pathway.^[24]^ Collectively, the above signaling pathways may potentially be new therapeutic targets for PE; however, the underlying mechanism requires further investigation.

Importantly, we identified CYBB, MAPK8, VEGFA, CCL2, and HMOX1 as the 5 most significant hub genes by constructing a PPI network and analyzing it using MCODE and 9 CytoHubba topology methods. *CYBB* and *MAPK8* were selected based on the diagnostic value of the ROC curve and the relationship between their expression levels and clinicopathological parameters. Cytochrome B-245 Beta Chain (also known as *gp91phox* and *Nox2*) is a protein-coding gene that mediates the mitochondrial electron transport chain and NADPH oxidases complex.^[25]^ CYBB-mediated ROS generation causes apoptosis through enhanced lipid peroxidation that destroys the inner mitochondrial membrane containing unsaturated fatty acids.^[26]^ This study found that the CYBB expression in histological sections was significantly lower in PE tissues than in normal samples. The AUC of CYBB was 0.767 which indicated a moderate diagnostic value for PE, and that CYBB expression negatively correlated with the uterine PI. Recent studies similarly indicated that CYBB promotes liver tumor formation in nonalcoholic steatohepatitis by inducing oxidative DNA damage.^[27]^ CYBB is a potential biomarker or target for the accurate diagnosis and treatment of gastric cancer.^[28]^ Mutations in the CYBB can lead to reduced NADPH oxidase activity and chronic granulomatous disease.^[29]^ However, the role and mechanisms related to OS in CYBB in PE require further study. Mitogen-activated protein kinase 8 (MAPK8) belongs to the MAP kinase family and is involved in various processes such as proliferation, migration, transcription regulation, and development.^[30]^ Our results indicated that MAPK8 mRNA expression significantly decreased in the placental tissues of PE patients compared to healthy samples. Significantly, *MAPK8* downregulation was negatively associated with the uterine PI, with an AUC of 0.764. This indicated that MAPK8 is a potential biomarker with moderate diagnostic value in PE. These results follow a study on early-onset PE.^[31]^ However, the mechanism by which MAPK8 regulates OS in PE requires further investigation.

This is the first study demonstrating that *CYBB* and *MAPK8* could be new biomarkers for PE. A drug–gene interaction network analysis showed that CYBB could be targeted by the apigenin, chrysin, and luteolin drugs. MAPK8 can also be targeted by tanzisertib and brimitol drugs.

Our study has several limitations. Firstly, this study was a retrospective study based on public datasets, and inherent selection bias may have affected its robustness. Secondly, further large-scale prospective studies are required to validate the clinical value of this hub gene signature. Also, additional experimental verification of placental tissues should be performed using quantitative real-time polymerase chain reaction (qRT-PCR), immunohistochemistry, and western blotting. In addition, complementary in vivo and in vitro experimental studies are necessary to reveal the potential molecular mechanisms of hub genes in PE to confirm our findings.

In conclusion, we demonstrated numerous key genes (VEGFA, CCL2, MAPK8, HMOX1, and CYBB) and possible molecular mechanisms associated with OS in the development and progression of PE. Our findings help elucidate the molecular mechanisms of OS underlying PE progression and provide a valuable basis for conducting further diagnostic and therapeutic research in patients with PE. However, further validation and the molecular mechanisms of these key genes should be looked into in the future.

## Author contributions

**Data curation:** Yueting Bai.

**Formal analysis:** Nianfeng Qian.

**Methodology:** Han Chen.

**Project administration:** Xuejing Liu, Hongqing Jiang.

**Software:** Shuo Wang, Chong Shen.

**Supervision:** Lina Wu.

**Validation:** Li Zhao.

**Visualization:** Chong Shen.

**Writing – original draft:** Xuejing Liu.

**Writing – review & editing:** Hongqing Jiang.

## Supplementary Material
